# Development of an Eye Tracking-Based Human-Computer Interface for Real-Time Applications

**DOI:** 10.3390/s19163630

**Published:** 2019-08-20

**Authors:** Radu Gabriel Bozomitu, Alexandru Păsărică, Daniela Tărniceriu, Cristian Rotariu

**Affiliations:** 1 Faculty of Electronics, Telecommunications and Information Technology, “Gheorghe Asachi” Technical University, Iaşi 700050, Romania; 2 Faculty of Medical Bioengineering, “Grigore T. Popa” University of Medicine and Pharmacy, Iaşi 700115, Romania

**Keywords:** detection rate, eye tracking, human computer interaction, image processing, open source software, pupil detection algorithms

## Abstract

In this paper, the development of an eye-tracking-based human–computer interface for real-time applications is presented. To identify the most appropriate pupil detection algorithm for the proposed interface, we analyzed the performance of eight algorithms, six of which we developed based on the most representative pupil center detection techniques. The accuracy of each algorithm was evaluated for different eye images from four representative databases and for video eye images using a new testing protocol for a scene image. For all video recordings, we determined the detection rate within a circular target 50-pixel area placed in different positions in the scene image, cursor controllability and stability on the user screen, and running time. The experimental results for a set of 30 subjects show a detection rate over 84% at 50 pixels for all proposed algorithms, and the best result (91.39%) was obtained with the circular Hough transform approach. Finally, this algorithm was implemented in the proposed interface to develop an eye typing application based on a virtual keyboard. The mean typing speed of the subjects who tested the system was higher than 20 characters per minute.

## 1. Introduction

The interest in eye detection applications has been considerably increasing [1]. Many eye detection methods are used in different applications such as neuroscience, psychology [2], assistive technologies to communicate with disabled patients [3,4,5,6], computer gaming, monitoring technologies for driver’s fatigue (in commercial and public transport) [7,8,9], in the advertising industry, person identification based on face recognition and eye (iris) detection [10,11], and in different military applications to help pilots aim weapons by simply looking at a target.

Eye tracking is the process of measuring either the point of gaze or the motion of an eye relative to the head. An eye tracker is a device for measuring eye position and eye movement [12,13,14]. The main eye tracking methods illustrated in the literature are based on a scleral search coil [15,16], infrared oculography (IROG) [15,17], electro-oculography (EOG) [15,18,19], and video oculography (VOG) [8,15,16,20,21]. Due to the progress in the field of electronics, gaze direction detection techniques have been developed in two basic directions: EOG [3] and digital image processing using VOG [4].

Different types of eye tracking systems have been reported in the literature [12,14,17,20,21,22,23]. Among these, head-mounted [23] and remote [20,24] eye tracking devices are used in real-time applications. One of the most commonly used applications of gaze-controlled interfaces is text entry using a virtual keyboard [5,25,26,27].

The increase in computing power has led to diversification of pupil detection algorithms (PDAs). The pupil detection algorithms presented in the literature can be classified into feature-based and model-based approaches [28,29].

Feature-based approaches involve detecting and localizing image features related to the position of the eye pupil. The best shape approximation of the eye pupil image provided by an infrared (IR) video camera is an ellipse. An ellipse can then be fit to the feature points using several techniques: least-squares fitting of ellipse (LSFE) [30,31]; voting-based methods, such as circular Hough transform (CHT) [32,33]; and searching-based methods, such as the random sample consensus (RANSAC) paradigm [34,35].

The LSFE algorithm performs the fitting of a conic (ellipse) to a set of data points by minimizing the sum of square algebraic distances of the feature points to the conic that is represented by the ellipse coefficients.

The CHT algorithm uses a parameter accumulator. Every edge pixel in the eye image votes for the parameters of all circles in which it can be a part. Maxima in the accumulator correspond to discovered circles [32]. Since a circle is completely described by three parameters, the circular Hough transform requires a three-dimensional (3D) accumulator.

The RANSAC algorithm is an iterative procedure that selects many small but random subsets of the input data, uses each subset to fit a model, and finds the model that best fits the input set of data points [34,35]. The algorithm determines the number of feature points (inliers) from the input data set that better approximates the ellipse model. This set of input points is called the consensus set. After a certain number of iterations, an ellipse is fit to the largest consensus set. The number of algorithm iterations can be reduced every time a new largest consensus set (represented by the percentage of inliers) is detected until the total number of inliers remains constant. Other feature-base approaches are represented by the projection method algorithm (PROJ) [36,37,38] and the curvature algorithm [39].

Model-based approaches do not explicitly detect features but rather find the best fitting model, which can be represented by either a circle or an ellipse, that is consistent with the eye image [29]. In the case of the Starburst algorithm [28,29], the result of ellipse fitting is improved through a model-based optimization using a Nelder–Mead Simplex search [29] that does not rely on feature detection. Starburst is a hybrid algorithm that combine both feature-based and model-based approaches. The algorithm uses the RANSAC paradigm to determine a set of candidate feature points.

Most studies presented in literature focused on the performance of different types of pupil detection algorithms on static eye images. Fuhl et al. [40] conducted a comparative study considering the Starburst [28,29,41], Świrski et al. [35,42], Pupil Labs [43], Sinusoidal Eye-Tracker (SET) [44], Exclusive Curve Selector (ExCuSe) [45,46], and Ellipse Selector (ElSe) [47] algorithms, but only for static eye images.

One of the most recent and accurate pupil detection algorithm in pervasive scenarios, Pupil Reconstructor (PuRe) [48], is based on novel edge segment selection and conditional segment combination schemes. To improve the detection rate and running time for real-time pervasive eye tracking, a new version, Pupil Reconstructor with Subsequent Tracking (PuReST), has been proposed [49].

Most of these algorithms are based on the principles mentioned above; the PDA proposed by Świrski et al. [35] is based on the RANSAC paradigm and ExCuSe, ElSe, PuRe and PuReST algorithms use the LSFE procedure after edge filtering by applying different morphologic operations.

In none of these studies was the real-time performance of PDAs in a scene image analyzed, many reporting a detection rate obtained only for raw eye images provided by the video camera. These studies focused on determining the algorithm accuracy when analyzing static eye images with low quality acquired in difficult conditions (bad illumination, occlusions from eyelashes, glasses reflections, etc.). Other algorithms reported in the literature provide a detection rate or an algorithm running time that are inadequate for real-time applications, as is the case for the algorithm based on new projection function [36].

To be used for eye tracking applications, the real-time performance of the PDAs must be investigated too. The experimental results illustrated in the literature for static eye images show that the accuracy of each pupil detection algorithm is strongly dependent on the characteristics of the eye tracking system used for acquiring these images.

In this paper, the development of an eye-tracking-based human–computer interface for real-time applications is presented. To find the best software solution for the proposed interface, we comparatively analyzed eight pupil detection algorithms based on the most representative state-of-the-art techniques for both static eye images and real-time applications using a novel testing protocol for evaluating the eye tracking system performance in the scene image. Of the eight PDAs analyzed in this study, we created six: three were developed in previous work in the field of eye detection, based on the CHT algorithm [50], LSFE method [51], and RANSAC paradigm [52], and the other three are newly proposed in this paper. The new three PDAs proposed in this study are based on the elliptical Hough transform (EHT), the new projection function method (PROJ), and the centroid method (CENT), as outlined in Section 2. The six algorithms proposed by the authors are compared with two well-known state-of-the-art open source algorithms: the Starburst algorithm [28,29,41] from the model-based approach category and the ExCuSe algorithm [45,46] from the category of edge filtering, which is based on oriented histograms calculated via the Angular Integral Projection Function [36]. The coarse pupil center estimation is then refined by ellipse estimation using the LSFE technique.

The real-time performance of each algorithm was evaluated using the head-mounted eye tracking interface developed by our team on a set of 30 subjects in different lighting conditions, resulting 240 distinct use cases, for a total of 370,182 eye images processed by the system.

This paper shows that the performance of any eye tracking system for real-time applications depends not only on the algorithm accuracy for raw eye images, but also on other factors, such as cursor controllability and stability on the user screen, running time, lighting conditions, the type of eye tracker device, and the user ability in using the system. According to these considerations, the algorithm based on circular Hough transform is shown to be best for real-time applications developed on the proposed eye tracking interface. As an application of this system, a low-cost eye typing application using a virtual keyboard is also presented.

The rest of this paper is organized as follows: Section 2 presents the hardware and software components of the proposed eye tracking interface, including our newly proposed PDAs, based on a new binarization procedure. Section 3 presents the experimental results of the analyzed PDAs obtained on the static eye images from four representative databases and the performance of the eye tracking interface based on these algorithms on the video eye images captured from a set of 30 subjects in laboratory conditions. The results obtained for each algorithm are comparatively discussed. In Section 4, a low-cost eye typing application developed on the proposed eye tracking interface is presented and Section 5 outlines the conclusions.

## 2. Materials and Methods

### 2.1. Video Oculography

Video oculography is a technique based on video analysis of eyeball movement using a video camera in the visible or infrared (IR) spectrum. The advantages of the methods based on the analysis of infrared eye images provided by video cameras include their versatility and being practically independent of lighting conditions and individual characteristics of the eye. The main disadvantage of infrared eye image capturing is that this technique cannot be used outdoors during daytime due to the ambient infrared illumination [28,29].

Infrared eye tracking applies either the bright-pupil technique (the pupil is clearly demarcated as a bright region due to the photo reflective nature of the back of the eye [29]) or dark-pupil techniques (the pupil is the darkest region in the image) [53]. The PDAs presented in this paper use the dark-pupil technique and, due to their versatility and robustness, they can be easily adapted for different real-time applications of the abovementioned areas.

### 2.2. Hardware System for VOG Signal Acquisition

**Figure 1:**
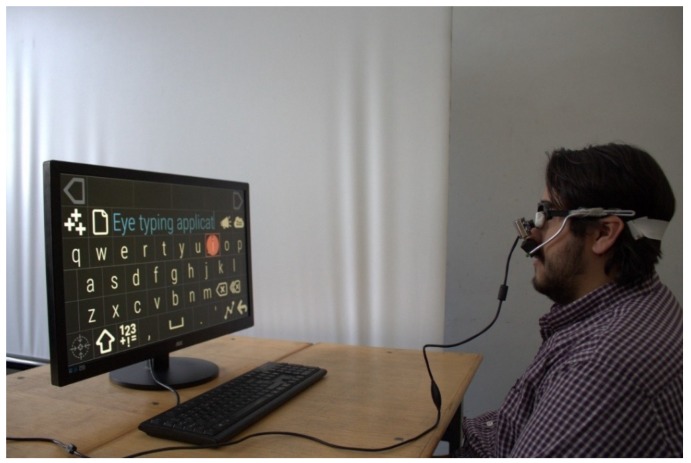
Head-mounted eye tracking interface.

The head-mounted eye tracking interface used in this study included an infrared video camera (model CVEHC-30H2, made in Guangdong China (Mainland)) with a resolution of 640 × 480 pixels (px) mounted on frame glasses immediately underneath the eye (Figure 1), connected to a personal computer (PC) or laptop for eye pupil image acquisition and processing. The video camera was modified by replacing the light emitting diodes (LEDs) in visible spectrum with infrared LEDs and placing an IR filter on its lens. Due to the infrared illumination of the eye, the corneal reflection is strongly present in the eye images produced by our eye tracking interface. Therefore, detecting the pupil in the eye images affected by corneal reflection is the main challenge faced by the investigated algorithms.

### 2.3. Eye Tracking Algorithm for Real-Time Applications

To integrate gaze detection techniques into human–computer interfaces for real-time applications, it is necessary to implement widely available, reliable, high speed, and high accuracy pupil detection algorithms compatible with the hardware used by present-day computers.

The proposed eye tracking interface moves a cursor on the user screen in real-time according to the user gaze direction. To implement different real-time applications, the user screen is divided into many cells. Target objects are placed in each cell that can be selected by the user’s gaze direction, according to their will or need. A target object selection is performed by focusing the user gaze direction in its selection area for a certain dwell time, established according to the user ability in using the system. The dwell time must be chosen to avoid the Midas touch problem [14], which is the selection of unwanted ideograms/objects on the user screen followed by the user’s gaze direction. For real-time applications, the minimum size of the target object that can be precisely controlled by the user’s gaze direction must be determined.

The compound eye tracking algorithm used for real-time applications implemented in our eye tracking system includes the following main procedures, described by Bozomitu et al. [50] in detail: (1) real-time eye image acquisition, (2) system calibration, (3) real-time pupil center detection, (4) mapping between raw eye image and scene image, (5) ideograms and/or objects selection, and (6) algorithm optimization to stabilize the cursor position on the user screen using real-time filtering and high frequency spikes canceling from the PDA signals.

**Figure 2:**
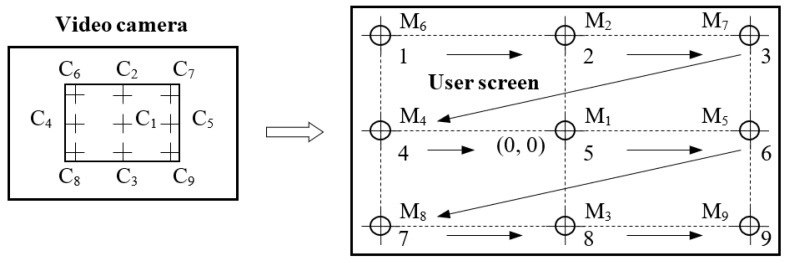
Mapping between eye pupil image and user screen cursor [50] (© 2015 IEEE).

The calibration stage is used to determine the mapping function coefficients and the radius of the eye pupil, required for optimal operation of the quantitative binarization procedure. The coefficients of the mapping function between the raw eye image and the scene image are obtained during the calibration process when a set of targets in known position (M*_i_*, *i* = 1, 2, …, 9; Figure 2) are displayed to the subject and the eye tracker position data are recorded, after a procedure illustrated by Stampe [54]. The radius of the pupil (required in the binarization stage) can be precisely determined, performing the CHT algorithm for the calibration point M_1_ placed in the center of the user screen.

Real-time pupil center detection is achieved in our eye tracking interface on the basis of eight PDAs (CENT, CHT, EHT, ExCuSe, LSFE, PROJ, RANSAC, and Starburst) to determine the most appropriate algorithm for real-time application.

The accuracy of the eye tracking system strongly depends on the precision of the calibration stage, during which the coefficients of the mapping function are determined. The convex shape of the eye causes nonlinear eye movement. Thus, the mapping function between the raw eye image provided by the IR video camera and the scene image is a user-dependent nonlinear function. Nonlinear mapping functions between the raw eye image and the scene image have the advantage of smooth changes in mapping across the user screen. The most common nonlinear function is biquadratic, introduced by Sheena and Borah in 1981 [55]. Bozomitu et al. [50] presented the mathematical calculation involved in the mapping technique in detail. The nonlinearity of the biquadratic mapping function is used to compensate for the nonlinearity of eye movement, so that the cursor, controlled by the user’s gaze direction, moves linearly across the screen.

To evaluate the PDA accuracy for real-time eye tracking applications, the mapping rate (MR) between the raw eye image and the scene image must be known. The MR depends on the calibration stage accuracy, the type and order of the mapping function used, the resolution of the user screen, and the user’s eye physiology. The mapping rate is defined as the radius of the circle resulting on the user screen when a circle with radius of 1 pixel is mapped between the raw eye image (with a resolution of 640 × 480 pixels) and the scene image (with full high definition (HD) resolution).

**Figure 3:**
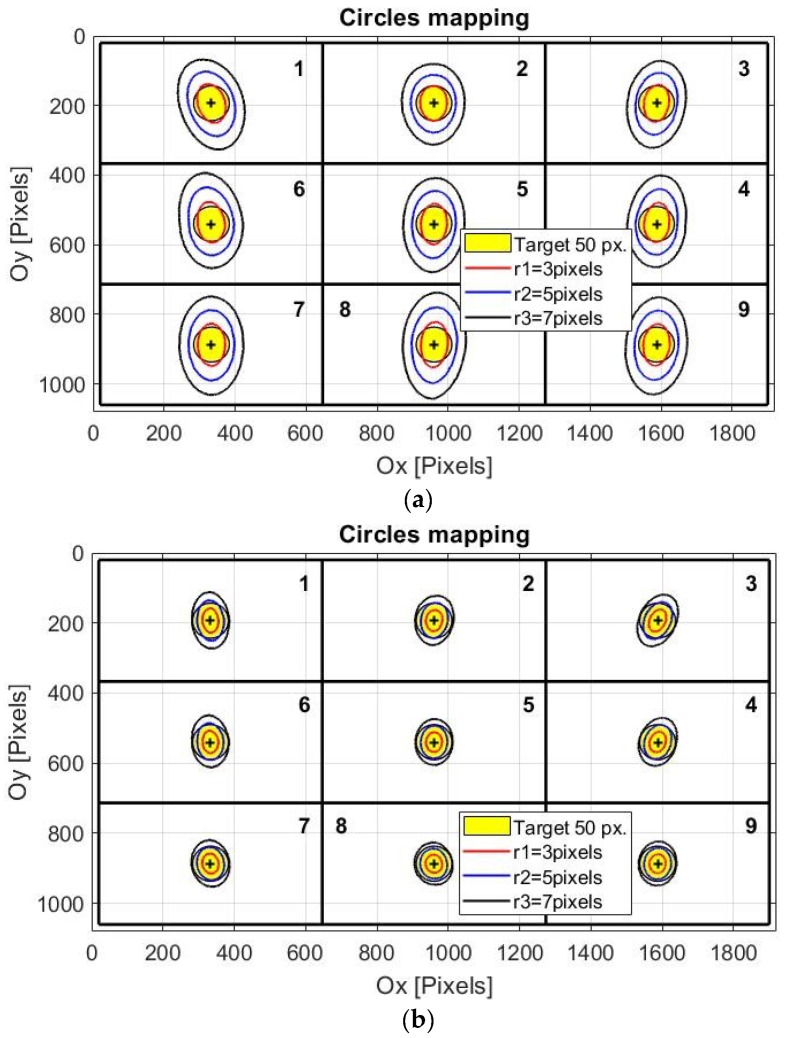
Mapping results on the scene image (user screen) for circles with radii of 3, 5, and 7 pixels in the raw eye image: (**a**) low accuracy of the calibration procedure (mapping rate (MR) = 15.96); (**b**) high accuracy of the calibration procedure (MR = 8.86).

Because the performance of any eye tracking interface depends on the calibration procedure quality, any eye tracking system must use an indicator of the accuracy of the calibration stage. This indicator can be determined by evaluating the system mapping rate during the calibration stage. Due to the nonlinearity of the mapping function, the mapping rate parameter must be calculated in different positions on the user screen. The best positions used to calculate the mapping rate are indicated by the nine identical quadrants on the user screen illustrated in Figure 3.

To calculate the MR of the proposed system, circles with radii *r* between 1 to 10 pixels are mapped between the raw eye image and the scene image, resulting in an irregular geometric shape that can be approximated by an ellipse or a circle. The radii of these circles in the raw eye image represent the algorithm accuracy in term of pixel error for any possible direction of the detection error.

Figure 3a,b present the evaluation of the calibration stage accuracy for two cases, corresponding to low and high accuracies of the calibration procedure, respectively. These figures show the resulting curves on the user screen when circles with radii of 3, 5, and 7 pixels in the raw eye image are mapped in each quadrant center on the user screen image. Coordinates of the points in the raw image are obtained by applying the inverse mapping function to the points in the quadrant centers from the scene image.

**Figure 4:**
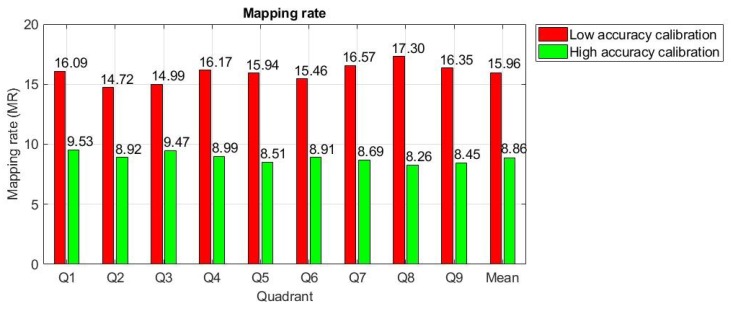
Mapping rate determination for low and high accuracies of the calibration procedure.

The shapes resulting on the user screen are not perfectly circular due to the nonlinearity of the mapping function used and an inaccurate calibration process. For simplicity, in the following analysis, these shapes are approximated by circles with radii *R_i_*(*r*), (*i* = 1, …, 9, corresponding to each quadrant, Q_1_, Q_2_, …, Q_9_) depending on mapping circle radius *r*. The mean value of the mapping rate for each quadrant in the scene image, *MR_i_*, illustrated in Figure 4 for low and high accuracies of the calibration procedure, were obtained by using the following equation:

(1) $$MR_{i}=\bar{R_{i}(r)/r},\;i=1,\;2,\;\ldots ,9,\;r=1,\;2,\;\ldots ,10$$

Thus, the mapping rate of the proposed eye tracking system can be approximated as the mean value of mapping rates for each quadrant, as follows:

(2) $$MR=\bar{MR_{i}},\;i=1,\;2,\;\ldots ,9$$

The radius *R*(*r*) of the circle that approximate the shape resulted on the user screen when a circle with radius *r* is mapped between the raw eye image and the scene image can be approximated by the following linear function:

(3) R(r)=MR×r

The MR is calculated for each subject during calibration stage and represents an important indicator of the calibration stage accuracy. The experimental results for 30 subjects in laboratory conditions showed that the MR for the propose system varies between 8.86 (the best calibration procedure) to 15.96 (the worst calibration procedure). Values higher than 16 for this parameter resulted in a decrease in the system accuracy for real-time applications and are not allowed during the calibration stage. For these situations, the calibration stage was resumed until a value less than 16 was obtained for MR.

The MR is also an important indicator of the expected algorithm accuracy in real-time applications in terms of target area size in the scene image, which can be precisely controlled by the user’s gaze direction. Thus, the expected algorithm accuracy in the scene image for real-time applications can be calculated using Equation (3). According to Equation (3), for an algorithm accuracy of 5 pixels and *MR* = 9, a circular target area with a radius of 45 pixels can be precisely controlled on the screen by the user’s gaze direction. This size of the target object is in agreement with the keypad dimensions of the virtual keyboard developed by OptiKey [56], which is used in the eye typing applications illustrated in Section 4.

In Section 3.2, the accuracy of the eye tracking system for real-time applications is evaluated using a target area with a radius of 50 pixels, placed in each quadrant center (illustrated in yellow in Figure 3). The size of the target area in the scene image for real-time applications depends on the PDA performance in terms of accuracy, cursor controllability and stability on the screen, running time, and the user’s experience in system operation.

### 2.4. New PDAs for Eye Tracking Applications

Generally, the six PDAs we propose (CENT, CHT, EHT, LSFE, PROJ, RANSAC) include the following stages: (1) eye image acquisition by using an IR camera, (2) eye image filtering to diminish the shot noise and line noise, (3) eye image binarization, (4) pupil reconstruction in the eye image, and (5) pupil center detection using different methods implemented in the analyzed PDAs. All these PDAs are based on a new adaptive quantitative binarization stage, suitable for real-time applications, which is described in the following.

#### 2.4.1. New Adaptive Quantitative Binarization Stage

Most of the PDAs reported in the literature are based on the binarization technique, implemented using different threshold-based selection methods for eye image segmentation with fixed or global/local adaptive threshold; a detailed study of different binarization techniques has been previously reported [57]. After binarization, different artifacts in the eye image may remain, which affect the pupil detection process. To solve this binarization issue (e.g., salt-and-pepper noise), our six proposed algorithms use a new binarization procedure based on the adaptive quantitative segmentation method [58]. The pupil detection is then performed by removing/diminishing the noise artefacts from the binarized eye image using a technique based on maximum area determination, followed by pupil reconstruction.

Unlike other algorithms reported in the literature that only determine candidates with pupil diameter within a certain range, PDAs based on the binarization procedure precisely detect the pupil, regardless of its size and position in the eye image. This is an important advantage, especially for real-time eye tracking applications where the pupil position and size may vary.

The new binarization procedure proposed in this paper uses an adaptive quantitative threshold and is based on the observation that, in the binarized eye image, the ratio between the number of object pixels (that correspond to the eye pupil) and the background pixels is almost constant for each subject if a head-mounted eye tracking interface is used. The percentage of the pixels that belong to the pupil of the total pixels from the eye image is given by the percentile value:

(4) $$t=\frac{\mathrm{pupil}\;\mathrm{image}\;\mathrm{size}}{\mathrm{eye}\;\mathrm{image}\;\mathrm{size}}\times 100\;(\%)$$

where eye image size is expressed in pixels^2^ and $\mathrm{pupil}\;\mathrm{image}\;\mathrm{size}=\pi r_{pupil}^{2}$ (the pupil being approximated by a circle of radius *r_pupil_*).

In the calibration stage, the pupil radius (*r_pupil_*) of each subject is determined using an algorithm based on the circular Hough transform. By using the determined pupil radius value in Equation (4), the optimum percentile value *t*_0_ is determined for each subject using the system. Therefore, for each image provided by the video camera, an optimum global threshold value *T* is determined by the algorithm using the percentile value *t*_0_. Thus, the threshold *T* indicates the maximum grey level from the eye image histogram over which *t* > *t*_0_. As a consequence, the performance of the binarization stage is not influenced by lighting conditions.

#### 2.4.2. Pupil Reconstruction in the Eye Image

**Figure 5:**
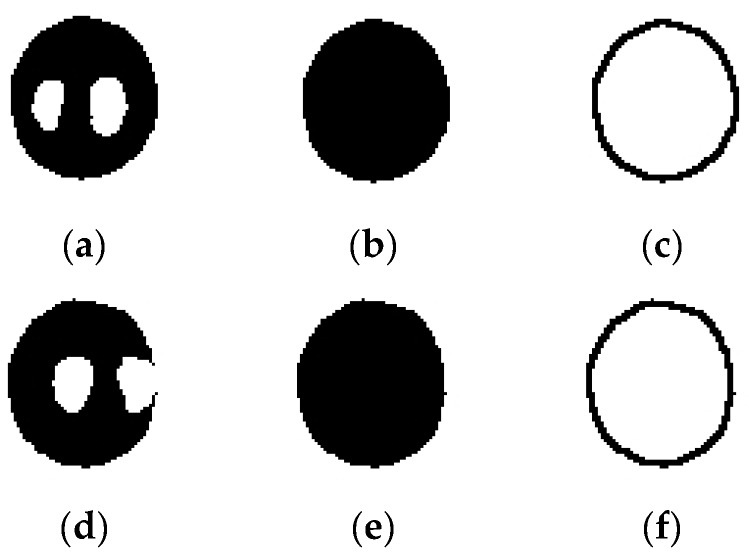
Pupil reconstruction stages when the corneal reflection is placed inside the pupil area and when it is located on the pupil edge: (**a**,**d**) eye pupil images after the binarization stage; (**b**,**e**) morphological reconstructions by dilation, filling the gaps due to corneal reflection and erosion; and (**c**,**f**) pupil contour detection.

The eye image resulted after binarization stage is affected by noise artefacts and corneal reflection. Pupil reconstruction in the eye image consists in the following techniques: (1) Removing/diminishing the noise artefacts. This stage is performed for all proposed algorithms except Hough-transform-based approaches. The noise artefacts due to false object pixels detection, which remain after the binarization stage, are removed/diminished from the background of the eye image using a technique based on maximum area determination. After the binarization stage, the CENT, LSFE, PROJ, and RANSAC algorithms find the connected components in binary eye image and the maximum area determined object is attributed to the eye pupil. Thus, the accuracy of these algorithms depends on the optimum choice of the percentile value *t*_0_. (2) Morphological reconstruction of the pupil. Many techniques have been used for pupil reconstruction in eye image processing in the literature: morphological open operation, which closes small bright gaps in the otherwise dark pupil region without significantly affecting the pupil’s contour [35] or linear interpolation in the case of Starburst algorithm, used to eliminate the corneal reflection (indicated in white in Figure 5a,d).

For all proposed algorithms, the morphological reconstruction of the pupil consists of image dilation, filling the gaps due to corneal reflection and image erosion. The dilation and erosion operation are both performed using circular structuring elements of the same size. After the binarization stage, the detected pupil may contain corneal reflection placed inside its area (Figure 5a) or located on its edge (Figure 5d). The resulted pupil images, after morphological reconstructions for both cases from Figure 5a,d, are illustrated in Figure 5b,e, respectively. Using this technique, the corneal reflection is completely eliminated for both cases illustrated in Figure 5a,d, and, as a consequence, the accuracy of the pupil center detection significantly increased. The detected eye pupil contours for both cases analyzed are shown in Figure 5c,f, respectively.

In the case of PDAs based on Hough transform (CHT and EHT algorithms), the maximum area object is no longer determined after the binarization stage. After the binarization and morphological processing stages, the edge detection is performed using the second order derivative of the resulting image. This is completed by combining smoothing and edge detection with the Laplacian of Gaussian (LoG) filter. The circular/elliptical Hough transform is then applied to the edge image to find the geometric shape (circle/ellipse) best fitted to the eye pupil. Thus, unlike other PDAs, the Hough-transform-based algorithms are less sensitive to the percentile value used in the binarization stage, and the pupil can be well detected under noise conditions, as is the case for many real-time applications. CHT and EHT algorithms operate well in nonuniform and variable illumination conditions, which is an important advantage for real-time applications, as shown in Section 3.

After detecting and reconstructing the pupil in the eye image, its center can be determined using different techniques implemented in the proposed algorithms. The simplest technique for detecting the pupil center is based on the centroid method algorithm (CENT), which involves determining the center of gravity of the pupil shape after binarization and pupil reconstruction stages.

Although very fast, the main drawback of this algorithm is that it is sensitive to different artifacts that remain after binarization and pupil reconstruction stages. The CENT algorithm does not detect the contour of the pupil, which may be a disadvantage for some applications. To solve these issues, we introduce the following two algorithms.

#### 2.4.3. EHT-Based Algorithm for Pupil Center Detection

The new EHT-based algorithm is introduced to solve the limitations of the CHT algorithm for eye images with the pupil placed at the edge of the sclera, where its shape is elliptical, and for rotated ellipse-shaped pupils. For these types of eye images, the EHT algorithm uses a five-dimensional (5D) accumulator, which corresponds to all geometric parameters (*x_c_*, *y_c_*, *a*, *b*, and *τ*) of an ellipse. Pupil center detection is more accurate than the CHT algorithm, but it is computationally expensive.

**Figure 6:**
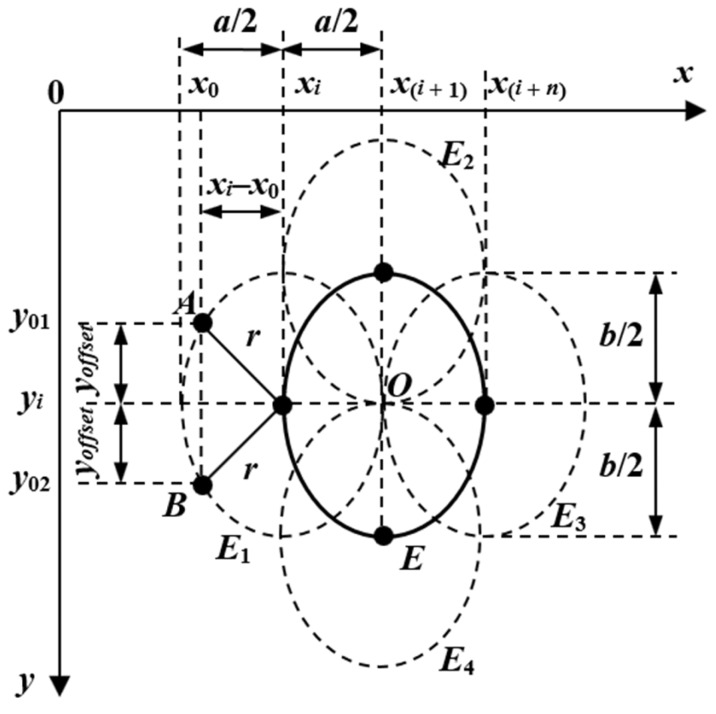
Elliptical Hough transform principle.

Each pixel in the image space correspond to an ellipse in the Hough space and vice versa. Thus, all points of the ellipse *E* in the image are mapped in several ellipses (*E*_1_, *E*_2_, *E*_3_, *E*_4_, …) having the same parameters. The intersection of these ellipses, denoted *O* in Figure 6, represents the center of the detected ellipse. Thus, the equation of the resulted ellipse *E*_1_ in Figure 6 can be written as:

(5) $$\frac{{\left(x-x_{i}\right)}^{2}}{a^{2}}+\frac{{\left(y-y_{i}\right)}^{2}}{b^{2}}=1$$

where (*x_i_*, *y_i_*) represents the coordinates of the ellipse *E*_1_ center and *a* and *b* are the two axes of the ellipse.

The two symmetrical points A and B on the ellipse *E*_1_ in Figure 6 have the coordinates (*x*_0_, *y*_01_) and (*x*_0_, *y*_02_), respectively. The coordinates on the *Oy* axis of these two points can be expressed as:

(6) $$\left\{ \begin{array}{l} y_{01}=y_{i}-y_{offset}\\ y_{02}=y_{i}+y_{offset} \end{array}\right.$$

The offset *y_offset_* on the *Oy* axis which results using Equation (6) in the ellipse in Equation (5) is:

(7) $$y_{offset}=b\times \sqrt{1-\frac{{\left(x_{i}-x_{0}\right)}^{2}}{a^{2}}}$$

Thus, this procedure can be used to detect the coordinates of the resulting ellipse points. The result of the Hough transform is stored in a table cell of the image size named the Hough accumulator. The accumulator value is updated for each ellipse generated using the elliptical Hough transform. Thus, the accumulator terms having the address provided by the points on the Hough ellipses (*E*1, *E*2, *E*3, *E*4, …) are increased by one unit. Thus, after a single pass through the image, the address (*x_c_*, *y_c_*) of the maximum value stored in the accumulator represents the center *O* of the detected ellipse *E*. To detect the best geometric parameters of the ellipse fitted to the pupil, the maximum value stored in the accumulator is calculated for a certain number *N* of (*a*, *b*, *τ*) triplets. The address of the maximum value stored in the accumulator after *N* passes through the image represents the coordinates of the optimum detected center of the ellipse. A high number of (*a*, *b*, *τ*) triplets processed by the algorithm significantly increases the algorithm accuracy, but increases its running time.

To decrease the EHT algorithm running time to ensure the minimum of 10 frames/s required for real-time applications, the angle *τ* of the ellipse has been neglected in the analysis illustrated in Section 3.2 and a four-dimensional (4D) accumulator was used.

#### 2.4.4. Projection Function Algorithm for Pupil Center Detection

**Figure 7:**
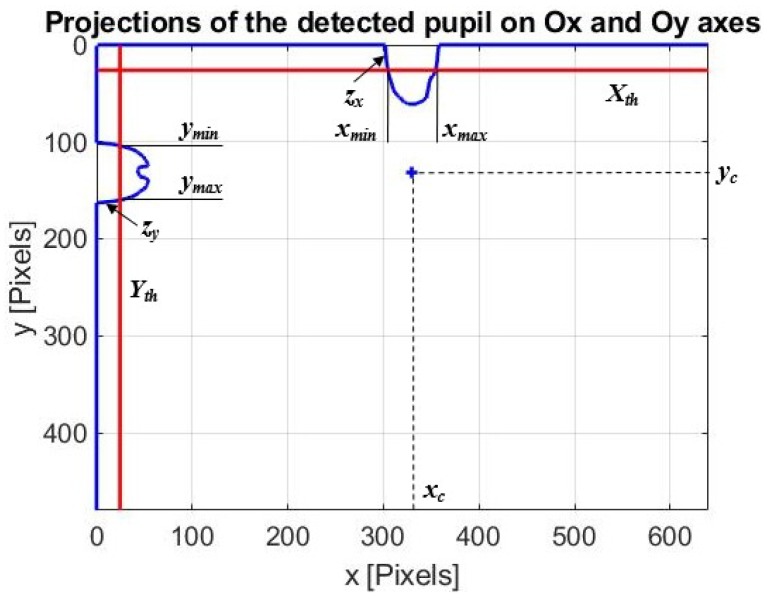
Principle of projection function method algorithm.

The new simplified projection function method algorithm (PROJ) proposed in this paper determines the projections of the binarized eye pupil image on the two axes of the coordinate system (Figure 7). The algorithm is very fast. If the binarized eye pupil image does not contain noise artifacts, the resulting projection vectors *z_x_* and *z_y_* on *Ox* and *Oy* axes, respectively, have strictly positive values only for the indices corresponding to the pupil shape, and values of 0 outside this range (Figure 7). False feature points detection, which cannot be totally avoided, determines the values different from 0 for projection vectors indices outside the pupil shape. To avoid the influence of these false pixels that belong to the background of the image, when computing the center of the pupil, an adaptive threshold value is used related to the elements of projection vectors to eliminate all false detections.

The threshold values, *X_th_* and *Y_th_*, plotted by a red line in Figure 7, can be written as:

(8) $$\left\{ \begin{array}{l} X_{th}=\frac{1}{m}\times \sum_{i=1}^{m}z_{x_{i}}(z_{x_{i}}>0)\\ Y_{th}=\frac{1}{n}\times \sum_{i=1}^{n}z_{y_{i}}(z_{y_{i}}>0) \end{array}\right.$$

where *m* and *n* are numbers of strict positive elements ($z_{x_{i}}$ and $z_{y_{i}}$) of projection vectors *z_x_* and *z_y_* on *Ox* and *Oy* axes, respectively.

Thus, according to Figure 7, the pupil center position (*x_c_*, *y_c_*) is indicated by the middle range of the projection vector elements higher than threshold values (*X_th_* and *Y_th_*) on both axes of the coordinate system and can be expressed by:

(9) $$\left\{ \begin{array}{l} x_{c}=\left(x_{\min}+x_{\max}\right)/2\\ y_{c}=\left(y_{\min}+y_{\max}\right)/2 \end{array}\right.$$

## 3. Results

### 3.1. Experimental Results of the Analyzed PDAs on Static Eye Images

The accuracy of the analyzed PDAs on static eye images was tested considering four representative databases. The first dataset (DB_1_) consists of 400 infrared eye images with a resolution of 640 × 480 pixels, with eye pupils of different shapes placed on different positions on the sclera and multiple corneal reflections, captured with the head-mounted eye tracking interface developed by our team. The second dataset (DB_2_-CIL) includes 400 infrared eye images with a resolution of 640 × 480 pixels from the publicly available database Casia-Iris-Lamp [59]. The third dataset (DB_3_-SW) contains 600 eye images with a resolution of 620 × 460 pixels, highly off axis, with eyelashes as presented by Świrski et al. [42], obtained as a uniformly random subset of left and right eye videos from two people. The forth dataset (DB_4_-ExCuSe dataset XII) contains 524 eye images with a resolution of 384 × 288 pixels from ExCuSe dataset XII with bad illumination [46]. For all datasets, the reference for the analysis is the ground truth data, which contain the hand-labelled eye images.

Since for real-time applications, the determination of the pupil center is of interest, to evaluate the PDA accuracy, we used the pixel error, calculated as the Euclidean distance between the detected and the manually labeled center of the eye pupil.

**Figure 8:**
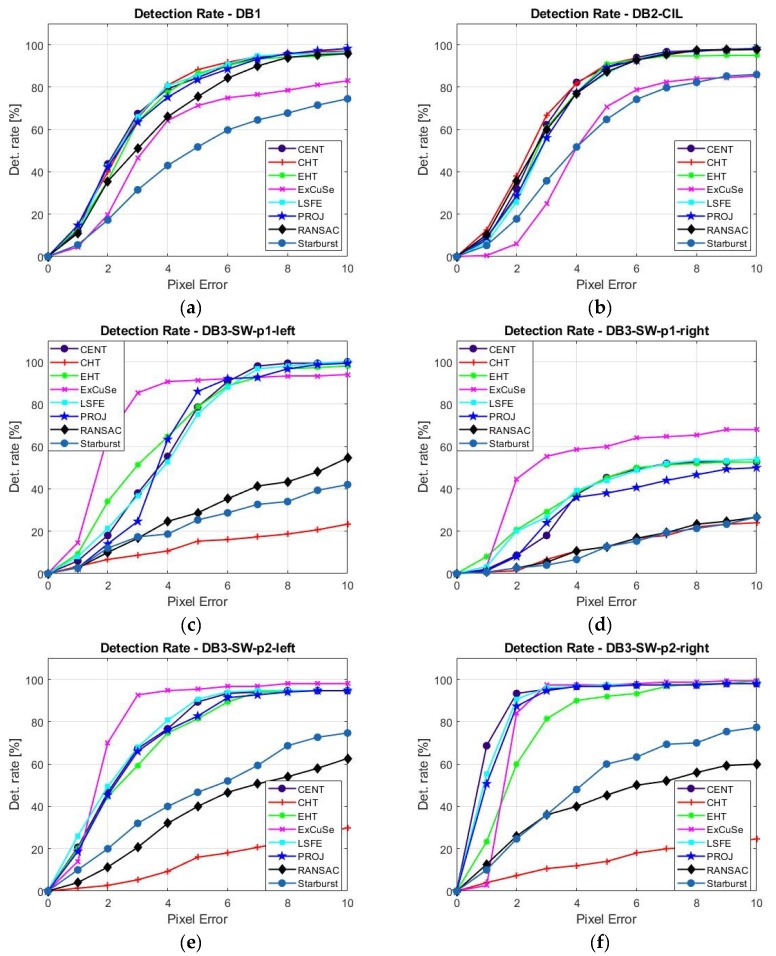

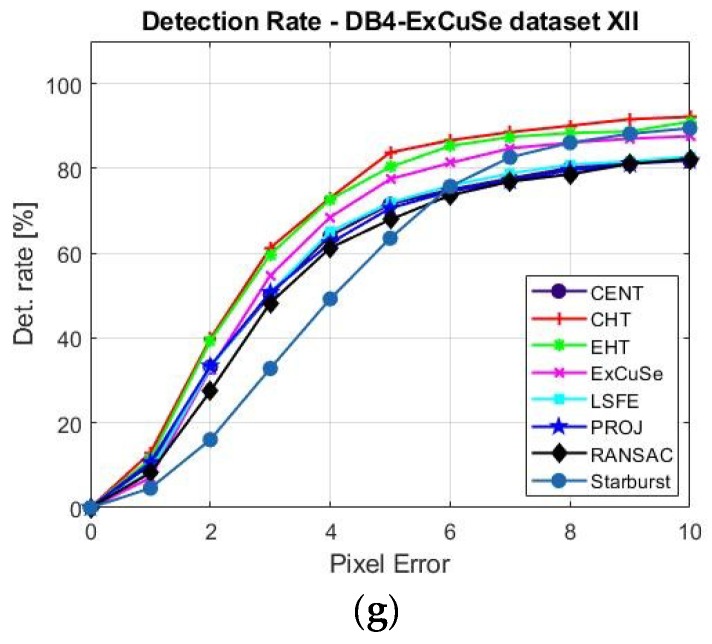
Detection rate vs. pixel error for all PDAs running on databases (**a**) DB_1_, (**b**) DB_2_-CIL, (**c**) DB_3_-SW-p1-left, (**d**) DB_3_-SW-p1-right, (**e**) DB_3_-SW-p2-left, (**f**) DB_3_-SW-p2-right, (**g**) DB_4_-ExCuSe.

Figure 8a–g present the detection rate depending on pixel error between 1 to 10 pixels for all studied algorithms determined for all four databases of eye images.

Table 1 illustrates the mean values ($\bar{d}$), standard deviations (*σ_d_*) of the pixel error, and the detection rate at five pixels (*DR*_5_), obtained by running each analyzed PDA on all four databases of eye images. The detection rate at five pixels represents the percentage of the eye pupil images for which the pixel error is lower than five pixels.

For the DB_1_ and DB_4_-ExCuSe databases, the best results for *DR*_5_ were produced by the CHT algorithm; for DB_2_-CIL, the best results were produced by both EHT and CHT algorithms; and for the DB_3_-SW database, the best results were produced by the ExCuSe algorithm.

For the DB_1_ and DB_2_-CIL databases, the lowest values of the *DR*_5_ were produced by Starburst and ExCuSe algorithms due to multiple corneal reflections present in these eye image datasets. For the DB_3_-SW database, the lowest results were produced by CHT, RANSAC, and Starburst algorithms due to the rotated ellipse shape of the pupil and its obstruction by eyelids and eyelashes, specific to these images. For the DB_4_-ExCuSe database, the lowest value of the *DR*_5_ was produced by the Starburst algorithm.

The CENT, EHT, ExCuSe, LSFE, and PROJ algorithms provided good results for static raw eye images (*DR_5_* ≥ 70%) for all databases, except for the DB_3_-SW-p1-right database.

For the eye images from the Świrski dataset DB_3_-SW-p1-right, a low detection rate was obtained for all PDAs (*DR_5_* ≤ 60%). These types of eye images are not suitable for real-time eye tracking applications due to these images being highly off axis and the pupil being covered with eyelids and eyelashes. This problem was solved by Świrski [35], where a detection rate over 87% on DB_3_-SW dataset (600 images) within an error threshold of five pixels is reported.

Although the CHT algorithm shows a low detection rate for DB_3_-SW database, it provides the highest detection rate for DB_1_, DB_2_-CIL, and DB_4_-ExCuSe databases and is the only algorithm of those tested that provides *σ_d_* ≤ 10 pixels for all datasets, except for the Świrski DB_3_-SW-p1-right dataset. The highest detection rate for most databases, the lower dispersion rate, and the high noise immunity for different lighting conditions make this algorithm an important candidate for real-time applications. The low detection rate of the CHT algorithm for DB_3_-SW database can be increased significantly using the new EHT algorithm proposed in this paper, which can better detect the rotated ellipse shape of the pupil, as shown by the results in Table 1. To evaluate the maximum performance of the EHT algorithm for static eye image, a 5D accumulator was used. Due to the significant increase in running time, for real-time applications, this algorithm uses only a 4D accumulator, as shown in Section 2.4.3.

The EHT algorithm is the only one of those tested that provides a *DR_5_* ≥ 75% among all datasets, except for the Świrski DB_3_-SW-p1-right dataset.

**Figure 9:**
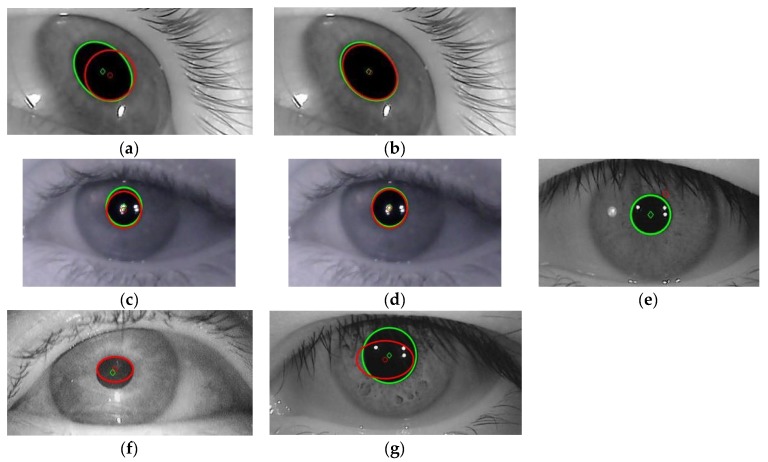
Typical detection errors of analyzed PDAs: (**a**) CHT, detection error due to elliptical shape of the pupil; (**b**) EHT, canceling the error introduce by CHT algorithm; (**c**,**d**) RANSAC, two different runnings on the same eye image with corneal reflection; (**e**) ExCuSe, loss of detection in an image affected by corneal reflection and occluded by eyelashes; and (**f**) PROJ and (**g**) LSFE, images sensitive to binarization stage (noisy eye image or pupil with occlusions). Legend: Green line—ideal pupil contour and center; red line—detected pupil contour and center.

Figure 9 illustrates some typical detection errors specific to the analyzed PDAs.

**Figure 10:**
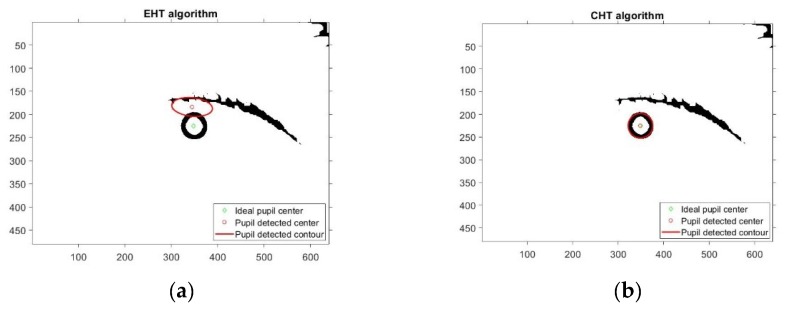
Results provided by EHT and CHT algorithms on the same noisy eye image and circular shape of the pupil: (**a**) failed detection for the EHT algorithm; (**b**) accurate detection for the CHT algorithm.

**Figure 11:**
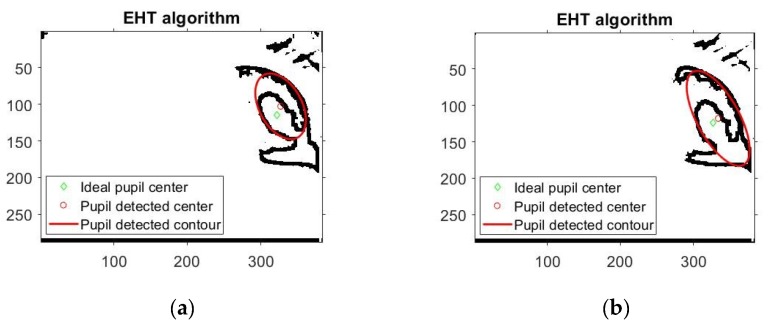
Typical detection errors provided by the EHT algorithm for noisy eye images.

Due to noisy eye images, there are many situations where the EHT based algorithm detects an ellipse rather than a circle. This case is illustrated in Figure 10 when the EHT algorithms fails and the CHT algorithm operates properly. Even for elliptical-shaped pupils, situations occur when the EHT-based algorithms fail, as shown by the experimental results illustrated in Figure 11. This explains the better results produced by the CHT algorithm on the DB_1_, DB_2_-CIL and DB_4_-ExCuSe databases.

The different results illustrated in Figure 8 and Table 1 show that the performance of each algorithm is strongly dependent on the type and particularities of the eye tracker device used for capturing the eye images from each database. Thus, the choice of the most appropriate pupil detection algorithm for a certain application depends on the type of eye tracking interface used to acquire the eye images.

To evaluate the performance of these algorithms for real-time eye tracking applications, many other factors may be considered. In Section 3.2, the performance of these algorithms for real-time applications are investigated, evaluating the accuracy of each algorithm in the scene image, cursor controllability and stability on the user screen, and running time.

### 3.2. Experimental Results of the Proposed Eye Tracking Interface for the Video Eye Images

In this section, the real-time performance of the proposed head-mounted eye tracking interface is analyzed. The subjects who tested the system in laboratory conditions were 30 students from the Faculty of Electronics, Telecommunications, and Information Technology from “Gheorghe Asachi” Technical University and Faculty of Medical Bioengineering from “Grigore T. Popa” University of Medicine and Pharmacy in Iaşi, Romania.

The inclusion criterion were the following: (1) students from the two faculties mentioned above who wanted to participate voluntarily in testing the eye tracking interface, (2) healthy subjects without any ocular disorder, (3) students who agreed to be trained on how to use the system, and (4) the ability to answer questions regarding the system usage.

**Figure 12:**
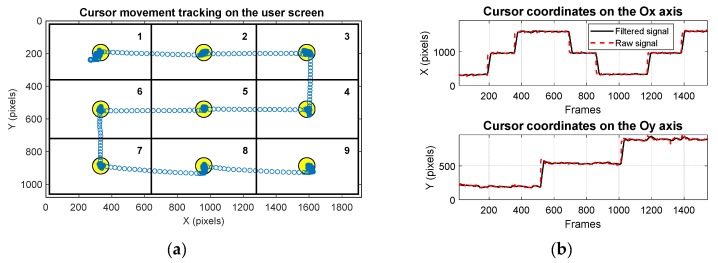
Real-time testing scenario: (**a**) cursor movement tracking on the user screen and (**b**) signals provided by the PDA on both axes of the coordinate system.

Participants were informed about the system’s operation and anonymity of the data collected in the experimental session. They were asked to keep their head in a stable position and look at the user screen placed at a distance of approximately 60 cm. The real-time testing scenario illustrated in Figure 12a involved moving the cursor by eye tracking over an imposed course on the user screen with full HD resolution while maintaining the cursor position as stable as possible in a circular target area within a radius of 50 pixels (illustrated in yellow in Figure 12a) placed in the center of each quadrant for a certain dwell time before moving to the next quadrant. Figure 12b presents the signals provided by the PDA for one recording during system testing, represented by the cursor coordinates on both axes according to the cursor movement tracking on the user screen.

Thus, the detected points on the user screen, corresponding to each quadrant, are known. Since the performance of any eye tracking system used for real-time application is influenced to some degree by the subjectivity of the users, its accuracy must be appreciated on a large set of subjects using statistical methods. The eight algorithms were tested on a set of subjects described above, resulting in 30 video recordings of the eye movement for each PDA (for a total of 240 use cases recorded). All 240 use cases involved a total of 370,182 eye images processed.

To evaluate the performance of all studied PDAs in the scene image, we calculated the Euclidean distance (*d*) between the detected cursor position in a quadrant on the user screen and the center of the target circle placed in that quadrant for each frame provided by the IR video camera.

For real-time applications, the detection rate (DR) is the percentage of all points from a quadrant (which are related to the quadrant) on the user screen for which their Euclidean distance calculated from the center of the quadrant to the detected cursor position is lower than the radius of the target circle.

A similar parameter, cluster detection rate (CDR), can be calculated related to the Euclidean distance for cluster evaluation (*d_c_*) where *d_c_* represents the Euclidean distance between the detected cursor position in a quadrant on the user screen and the center of the cluster points in that quadrant. The coordinates of the cluster center are given by the mean value of the coordinates of the quadrant points on both axes of the coordinate system.

The cluster dispersion rate (CDIR) is the percentage of the detected points on the user screen for which their Euclidean distance for cluster evaluation is higher than the radius of the target circle. For percentage values: CDIR = 100–CDR.

For each subject *s* (*s* = 1, …, 30) who tested the system, the following parameters were determined in the scene image: the mean value ($\bar{d_{s}}$) and the standard deviation (*σ_s_*) of the Euclidean distance, the detection rate at 50 pixels (*sDR_50_*), and the cluster dispersion rate at 50 pixels (*sCDIR*_50_).

**Figure 13:**
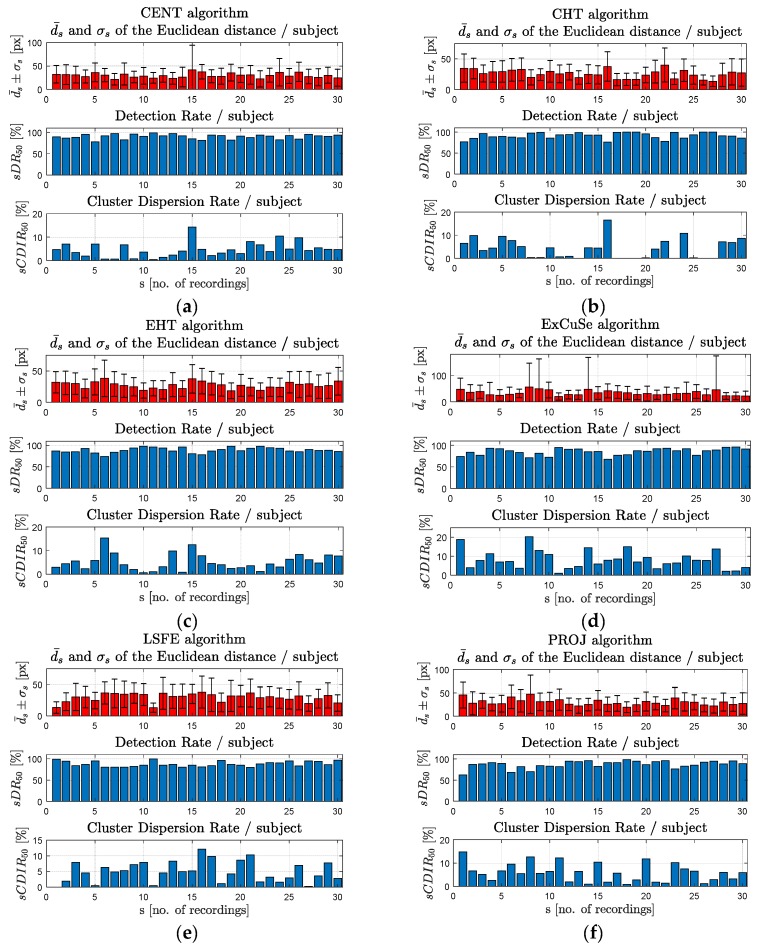

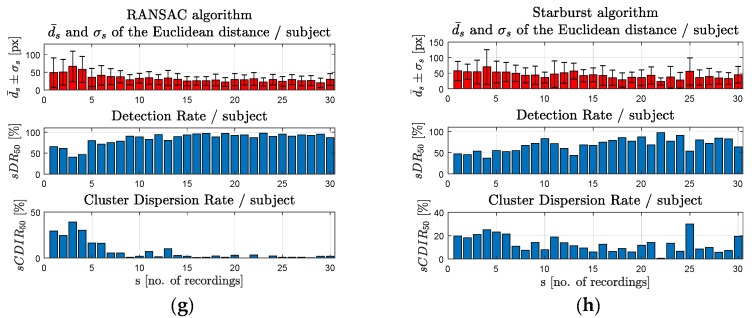
Experimental results obtained on the video eye images for the (**a**) CENT, (**b**) CHT, (**c**) EHT, (**d**) ExCuSe, (**e**) LSFE, (**f**) PROJ, (**g**) RANSAC, and (**h**) Starburst algorithms.

In Figure 13a–h, the results obtained for each subject are plotted for each PDA.

In Table 2 we synthetize the results obtained for each tested PDA by determining the overall mean value ($\bar{d}$) and the standard deviation (*σ*) of the Euclidean distance, and the DR at 50 pixels (*DR*_50_).

For each tested PDA, Table 3 presents the overall mean value ($\bar{d_{c}}$) and standard deviation (*σ_c_*) of the Euclidean distance for cluster evaluation, and the *CDIR* at 50 pixels (*CDIR*_50_).

Table 4 lists the algorithm running time for each tested PDA. These values were determined using a computer with I7 7700 K Intel processor at 4.2 GHz, with 8 Gb RAM, and SSD.

**Figure 14:**
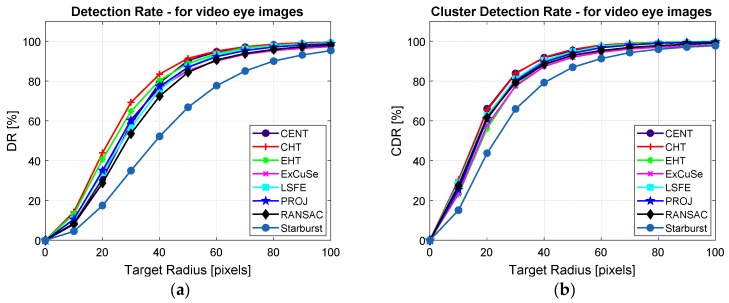
(**a**) Detection rate and (**b**) cluster detection rate depending on target area radius for all studied algorithms for real-time applications.

Figure 14a,b present the detection rate and the cluster detection rate, respectively, depending on a circular target with radius between 10 to 100 pixels obtained for all studied PDAs for a real-time scenario.

The results presented in Figure 14a indicate the positioning precision of the cursor on a circular target area with radii between 10 to 100 pixels. The results presented in Table 2 and Figure 14a show a detection rate at 50 pixels between 84.46% and 91.39% for CENT, CHT, EHT, ExCuSe, LSFE, PROJ, and RANSAC algorithms. The Starburst algorithm produced a lower detection rate of 66.86%.

The results in Table 3 and Figure 14b represent an indicator of the cursor stability, signifying the user’s ability to easily maintain the cursor in the same position on the screen to perform an ideogram selection.

Although the ExCuSe algorithm has a *DR*_50_ of 85.32%, it has a high dispersion rate (*CDIR*_50_ = 8.02%) and a longer running time (0.094 s), which means low controllability of the algorithm for real-time applications.

According to Table 2 and Table 3, the best results were obtained using the CHT algorithm, which provided the highest detection rate at 50 pixels (91.39%) and the lowest cluster dispersion rate (4.07%). Also, the CHT algorithm provided the lowest values for mean Euclidean distance and standard deviation of the Euclidean distance for real-time applications.

These results show that the proposed eye tracking interface based on the CHT algorithm is highly accurate, provides reliable controllability, and user-friendly, and thus can be a valid solution for different types of real-time applications, like communication over the Internet, text entry using a virtual keyboard (as illustrated in Section 4), computer gaming, virtual reality, and assistive technologies for communications with disabled patients [6].

### 3.3. Discussion

According to experimentally obtained results, some conclusions regarding the operation of the eye tracking system for real-time applications were drawn. Pupil detection algorithm based on least squares fitting of an ellipse is numerically stable, very fast, and precise. LSFE is the most commonly used principle in the pupil detection algorithms reported in the literature [39,45,47,48,49]. The eye pupil is approximated by an ellipse using a mathematical model of the ellipse, which determines the high stability of the cursor on the user screen. The precision of the algorithm is dependent on the performance of the binarization stage. It is influenced by the physiological parameters of the eye, dilation and contraction of the pupil depending on the illumination condition, and the angle of the gaze direction. The main disadvantage is in the case of outlier points, the detected ellipse may not be the best geometrical model of the eye pupil. This situation may occur in the case of noisy frames and nonuniform or fluctuating lighting conditions, when the feature input points provided by the LSFE algorithm are not best fitted to the pupil ellipse (Figure 9g). These situations, which can create significant errors in pupil center coordinate detection, have been significantly improved using the new binarization technique, proposed here.

The RANSAC paradigm-based algorithm provides high accuracy and moderate running time. Due to the classification of pixels on the pupil contour for inliers and outliers, the algorithm performs well with noisy frames and nonuniform lighting conditions. The RANSAC algorithm is based on randomly detecting the largest consensus set to draw an ellipse that better fits the eye pupil. Thus, the algorithm can produce different results regarding the coordinates of the detected pupil center for identical conditions of the algorithm running on the same eye images, as shown in Figure 9c,d. This produces a noise overlap on the signals provided by the PDA, resulting in cursor instability on the user screen, which makes this algorithm unsuitable for real-time applications.

Pupil detection algorithm using the circular Hough transform can operate in difficult lighting conditions, tolerates gaps in feature boundary descriptions, and best detects the pupil center in noisy frames. It is a robust algorithm that is based on detecting the circle that best approximates the pupil in the binarized eye image. The higher stability of the cursor on the user screen is due to the lowest dispersion rate for real-time applications (*CDIR*_50_ = 4.07%), which confirms the results obtained with the static eye images from all four datasets tested, for which *σ_d_* ≤ 10 pixels was achieved. The main drawback of this algorithm is that it always approximates the pupil image with a circle, but this is not always true. Due to the filming angle, when the pupil is placed at the edges of the sclera (left, right, top, bottom), the pupil image is no longer circular, so that the geometric shape, which better approximates the pupil in this case, is an ellipse. For this reason, since this algorithm always detects circles, it creates errors when detecting the pupil center, as shown in Figure 9a. Thus, the error of the pupil detected center depends on the pupil position on the sclera.

The elliptical Hough-transform-based algorithm is highly accurate for static eye images, can detect the elliptical shape of the pupil (Figure 9b), but the algorithm running time is significantly longer for real-time applications because the mathematical description of an ellipse requires five parameters compared to only three in the case of circle description. The experimental results showed that the performance of the EHT algorithm is worse than that of the CHT algorithm for different databases containing eye images with a circular pupil shape. This can be explained by both algorithms detecting the pupil center in noisy eye images. Thus, under these conditions, the EHT algorithm can detect elliptical shapes rather than circular shapes in the noisy eye image, which does not represent the eye pupil, as shown in Figure 10a and Figure 11. The EHT algorithm performs better than CHT algorithm for databases including eye images with a rotated ellipse shape of the pupil, as is the case for the DB_3_-SW Świrski database. These images are highly off axis due to the filming angle, which is not the case for the eye images provided by the eye tracking interface proposed in this paper. However, the main drawback of the EHT algorithm is that for increased accuracy, this algorithm requires a longer running time, which is not suitable for real-time applications. Increasing computation power will increase the attractiveness of this algorithm, but it will increase the price of the system.

Algorithms based on the projection method and centroid detection provide high real-time accuracy if no artifacts appear in the eye image. Both algorithms also provide high controllability and very fast running time. The precision of both algorithms is dependent on the performance of the binarization stage (Figure 9f). The accuracy of the projection method algorithm is dependent on the threshold values used for the two axes of the coordinate system. A low threshold value increases the algorithm accuracy, but decreases the noise immunity; high values for these parameters increase the noise immunity of the algorithm, but decrease its accuracy. Thus, a trade-off between accuracy and noise immunity for the projection method algorithm must be considered for real-time applications. The centroid method algorithm does not detect the contour of the pupil and its accuracy decreases if artefacts appear after the binarization procedure. We noticed a variation in the detected pupil center position between consecutive frames due to the random geometric shape of the maximum area detected by the algorithm as a result of the binarization stage.

The Starburst algorithm provides lower accuracy, lower controllability, and its operation is severely influenced by noisy frames and nonuniform lighting conditions. The pupil is approximated by an ellipse using randomly determined features points, which causes a high dispersion rate; this significantly affects the stability of the cursor on the user screen. The algorithm accuracy is dependent on the running time.

The ExCuSe algorithm is highly accurate, but has a long running time and high dispersion rate due to loss of detection on certain frames affected by the corneal reflection (Figure 9e). The eye tracking system based on this algorithm requires more user experience.

The best results produced by the CHT algorithm for real-time applications can be explained by some important features of the proposed eye tracing interface. The filming angle of the video camera was chosen to avoid the rotated ellipse shape of the pupil, which significantly improves the algorithm accuracy (*DR*_50_ = 91.39%). The CHT algorithm provides highest noise immunity, the lowest dispersion rate, and a reasonable running time. Given these properties, pupil detection using CHT is a technique suitable for algorithms that rely on binarization of the eye image. All these factors indicate that the CHT algorithm is the most user-friendly for real-time applications running on our designed eye tracking interface.

The major challenge in the research and development of new eye tracking applications is implementing new systems capable of operating properly both indoors and outdoors [60]. Future eye tracking systems will use new technologies and sensors that better mimic biological sensing and the eye structure. For example, the silicon retina has high potential for high speed eye tracking to provide robust detections in ambient light conditions [61]. As shown in Majaranta et al. [14], the pixels in the silicon retina are able to asynchronously respond to relative changes in intensity. This enables fast and robust detection of movement and tracking of objects in varying light conditions where traditional video- and IR-based eye tracking systems typically fail.

## 4. Implementing an Eye Typing Application Using the Proposed Interface

**Figure 15:**
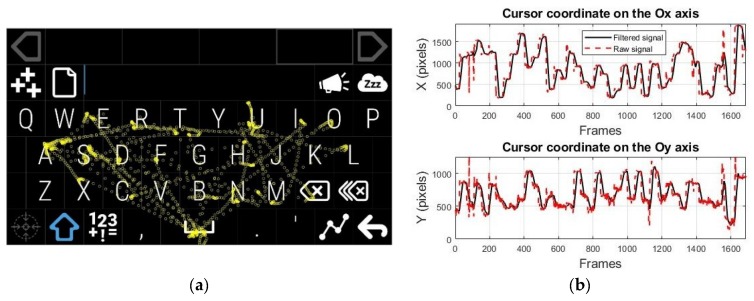
(**a**) Cursor movement tracking on the virtual keyboard developed by OptiKey [56] and (**b**) signals provided by the PDA on both axes of the coordinate system during typing a sentence.

To test the real-time performance of the proposed eye tracking interface for a real case scenario, in this section, we present an eye typing application using a virtual keyboard. The layout of the virtual keyboard is important for real-time applications [25]. In our application, we used an open source QWERTY virtual keyboard, developed by OptiKey from GitHub [56], as illustrated in Figure 15a. This keyboard provides support for dwell selection time and its layout allows the use of one keystroke per character (KSPC). Since our main purpose was to test the performance of the pupil detection algorithm (number of keystrokes in a time unit), the autocorrect function was not used. The size of keypads used by this keyboard is larger than 50 pixels, which represents the radius of the circular target objects used in our system design, illustrated in Section 3.2. The software solution used by the proposed eye tracking interface for implementing an eye typing application uses the CHT algorithm for pupil center detection, which proved to be the best for real-time applications, according to the tests results in Section 3.2.

The eye typing application was tested on the same set of 30 subjects, described in the previous section. Although all subjects were beginners in eye typing, they had some experience using the eye tracking interface gained when testing the system accuracy, as shown in Section 3.2.

Each subject typed five sentences using their gaze direction. Each block of five sentences contained an average of 135 characters. The chosen sentences represent Romanian translation of the Phrase Set proposed by MacKenzie and Soukoreff [62]. The subjects’ task consisted of typing the required sentences as fast and accurately as possible using only small letters. The dwell time was set to 1000 ms. The calibration stage was performed for each sentence from a block of five. To avoid fatigue, a five-minute break between the sentences was given. Because the participants are beginners in using the eye tracking system, they were provided one practice phrase (about 25 characters) prior to data collection [25]. Below is an example of a block with five sentences used to test the eye typing application: (1) my watch fell in the water, (2) prevailing wind from the east, (3) never too rich and never too thin, (4) breathing is difficult, and (5) I can see the rings on Saturn [62].

Figure 15a plots an example of the cursor movement tracking on the virtual keyboard layout for one record during system testing; Figure 15b represents the signals provided by the PDA on both axes of the coordinate system for the same record.

According to the taxonomy proposed by MacKenzie and Soukoreff [63], the following statistical indicators were calculated: total error rate (TER), not corrected error rate (NCER), and corrected error rate (CER). These parameters are calculated using the following equations, respectively [63]:

(10) $$\mathrm{Total}\;\mathrm{Error}\;\mathrm{Rate}=\frac{INF+IF}{C+INF+IF}\times 100\;(\%)$$

(11) $$\mathrm{Not}\;\mathrm{Corrected}\;\mathrm{Error}\;\mathrm{Rate}=\frac{INF}{C+INF+IF}\times 100\;(\%)$$

(12) $$\mathrm{Corrected}\;\mathrm{Error}\;\mathrm{Rate}=\frac{IF}{C+INF+IF}\times 100\;(\%)$$

where *C* is the number of correct inputs, *IF* is the number of errors made and corrected, and *INF* is the number of errors but not corrected [63].

**Figure 16:**
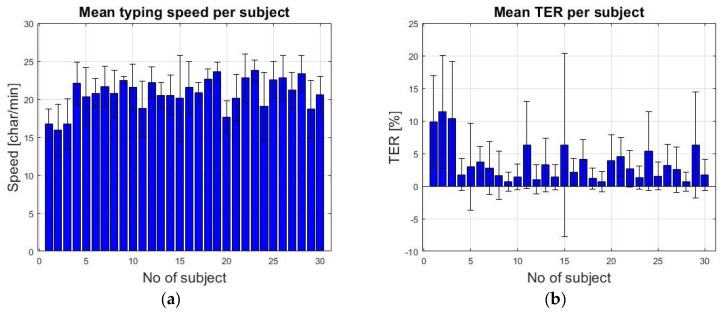
Mean value and standard deviation of the (**a**) typing speed per subject and (**b**) TER per subject.

For all subjects, the typing speed (characters/min) and the MR defined in Section 2 were calculated. For all 30 subjects, the interest parameters were calculated for each typed sentence, as well as the mean values for each block of five sentences. Figure 16 plots the mean value and standard deviation of the typing speed and TER for each subject who tested the system.

Table 5 lists the overall mean value and the standard deviation of all considered parameters recorded during the test session.

All subjects who tested the proposed eye tracking interface for the eye typing application were able to perform the calibration stage and accomplished 100% of their tasks. Of the 30 subjects who tested the application, three (10%) made only one mistake that was corrected, and one subject (3.33%) made one mistake that was not corrected. The greatest number of errors made during application testing was recorded by a single subject who made 19 errors, of which 14 were corrected and five were not. Many errors occurred due to inaccurate calibration at the beginning of the test session. To avoid the system being imprecisely calibrated, the eye tracking system proposed in this paper uses a calibration indicator, which is represented by the mapping rate parameter, calculated during the calibration stage. If the mapping rate is higher than a certain threshold, then the system failed to perform an accurate calibration procedure required for eye typing application, and, as a consequence, the subject must repeat the calibration stage. From practical experience, this threshold was set to 16. During the test session, only one subject needed to repeat the calibration stage. The best calibration procedure quality for a sentence was obtained for MR = 8.86 and the worst quality that was accepted was obtained for MR = 15.96, as shown in Figure 3 and Figure 4.

The experimental results with 30 novice subjects who used a constant dwell time of 1000 ms have shown a mean typing speed of about 20 characters per minute, which it is in line with the observations presented in Majaranta et. al. [64]. Taking into account that all 30 subjects have succeeded to use the system after a short training period, fulfilling their tasks, shows that the proposed system can be a valid solution for real-time eye tracking applications.

The typing speed of the proposed eye tracking interface can be significantly increased through more user training, by decreasing the dwell time value or by using different word and letter prediction techniques, as it is shown in [26] and [27].

At the end of the test session, the participants completed the System Usability Scale (SUS) questionnaire to determine the performance of the eye tracking interface based on CHT algorithm. Each item’s score contribution ranges from zero to four (with four being the most positive response). The SUS score is calculated according to the procedure previously illustrated [65].

The 10 statements on the SUS are as follows [65]:

(S1) I think that I would like to use this eye tracking interface frequently.
(S2) I found the eye tracking interface unnecessarily complex.
(S3) I thought the eye tracking interface was easy to use.
(S4) I think that I would need the support of a technical person to be able to use this eye tracking interface.
(S5) I found the various functions in this eye tracking interface were well integrated.
(S6) I thought there was too much inconsistency in this eye tracking interface.
(S7) I would imagine that most people would learn to use this eye tracking interface very quickly.
(S8) I found the eye tracking interface cumbersome to use.
(S9) I felt very confident using the eye tracking interface.
(S10) I needed to learn a lot of things before I could get going with this eye tracking interface.

**Figure 17:**
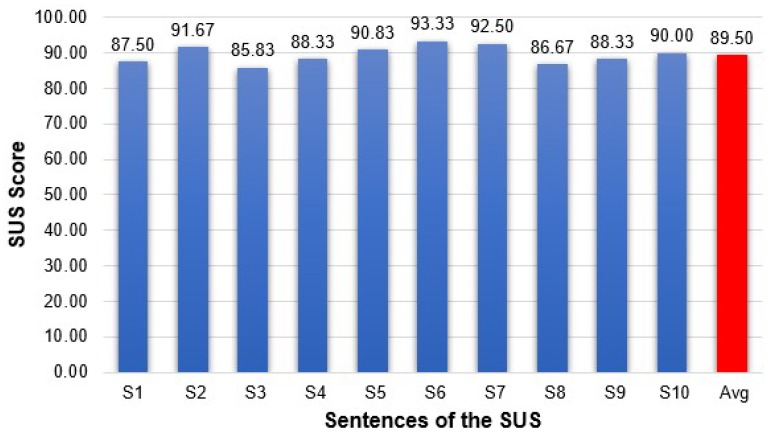
System Usability Scale (SUS) score of each item of the questionnaire.

Figure 17 provides the result of the SUS questionnaire score obtained for each item. Regarding the system usability, four subjects provided the maximum SUS score. The lowest score was 67.5. The overall mean score was 89.50, with a standard deviation of 6.87. Many subjects had difficulty using the system without training and getting accustomed to the head-mounted eye tracking interface because the subjects did not have prior experience with this type of system.

To improve this situation, the subjects received a practice phrase before starting the experiment. The average value of 89.50 indicates the good performance of the eye tracking system according to the scoring system established in specialty literature [66].

## 5. Conclusions

In this study, we developed an eye tracking-based human–computer interface for real-time applications. The most suitable algorithm for the eye tracking interface was determined by analyzing the performance of different PDAs on static and video eye images.

To evaluate the algorithm accuracy for video eye images, we proposed a new method to determine the mapping rate between the raw eye image and the scene image. Using this method and knowing the algorithm accuracy for static eye images, we determined the algorithm accuracy expected for real-time applications as the minimum size of the target area on the user screen, which can be precisely controlled by the user’s gaze direction. We showed that the high precision of a PDA on static eye images does not guarantee high performance for video eye images. To ensure high performance for real-time applications, other parameters, like cursor controllability and stability on the user screen and algorithm running time, were also considered.

Experimental sessions involved testing the eye tracking interface that uses all eight PDAs on a set of 30 subjects in laboratory conditions using a new testing protocol developed using scene images for different real-time operating scenarios and lighting conditions. The resulting 240 use cases were statistically processed.

The experimental results obtained by the proposed PDAs (CENT, CHT, EHT, LSFE, PROJ, and RANSAC) produced detection rates between 84.46% and 91.39% in the scene image for a target area radius of 50 pixels on the user screen. The best results in term of accuracy and cursor controllability and stability on the user screen were achieved using the CHT-based algorithm, which was the most user-friendly for the developed interface.

The performance of the proposed low-cost eye tracking interface based on the CHT algorithm was evaluated on an eye typing application using an on-screen virtual keyboard. The mean typing speed obtained from a set of 30 subjects was 20.74 characters/min with a mean total error rate of 3.55%.

Considering the low price, high accuracy for variable and non-uniform lighting conditions, and the results of the SUS questionnaire, the proposed eye tracking interface can be a valuable solution for different types of real-time applications. Depending on the application type and the device used, a trade-off between accuracy, controllability, running time, noise sensitivity, and performance of the PDA in nonuniform lighting conditions must be considered in any eye tracking system design.

## Figures and Tables

**Table 1:** Performances of the PDAs for static eye images from all four databases.

PDA		CENT	CHT	EHT	ExCuSe	LSFE	PROJ	RANSAC	Starburst
DB_1_ (400 images)	$\bar{d}$ **(px)**	2.96	2.99	5.35	7.94	3.01	2.98	3.61	19.61
	***σ_d_* (px)**	2.48	2.42	23.10	21.44	2.54	2.36	2.90	35.12
	***DR*_5_ (%)**	84.50	88.25	86.50	71.25	85.50	83.50	75.50	51.75
DB_2_ -CIL (400 images)	$\bar{d}$ **(px)**	3.00	3.01	4.18	8.61	3.23	3.12	3.04	5.32
	***σ_d_* (px)**	2.35	3.04	10.17	16.72	2.34	2.31	2.25	4.86
	***DR*_5_ (%)**	89.50	90.75	91.00	70.75	86.50	89.25	87.25	64.75
DB_3_ -SW-p1-left (150 images)	$\bar{d}$ **(px)**	3.66	14.78	3.41	3.08	3.74	3.79	9.61	21.85
	***σ_d_* (px)**	1.72	7.28	2.57	5.31	1.87	1.69	5.97	31.10
	***DR*_5_ (%)**	78.66	15.33	78.66	91.33	75.33	86.00	28.66	25.33
DB_3_ -SW-p1-right (150 images)	$\bar{d}$ **(px)**	76.31	55.51	61.86	30.21	74.25	76.97	63.56	27.56
	***σ_d_* (px)**	88.95	81.27	88.48	64.63	87.19	88.56	88.27	26.53
	***DR*_5_ (%)**	45.33	12.66	45.33	60.00	44.00	38.00	12.66	12.66
DB_3_ -SW-p2-left (150 images)	$\bar{d}$ **(px)**	8.87	15.10	8.72	2.10	8.70	9.05	15.26	7.91
	***σ_d_* (px)**	32.51	7.75	31.99	2.45	32.51	32.53	32.42	9.74
	***DR*_5_ (%)**	89.33	16.00	81.33	95.33	90.66	82.66	40.00	46.66
DB_3_ -SW-p2-right (150 images)	$\bar{d}$ **(px)**	3.97	15.99	4.75	1.87	4.04	4.22	30.20	6.60
	***σ_d_* (px)**	34.10	8.07	32.33	1.68	33.39	34.01	87.95	6.45
	***DR*_5_ (%)**	96.66	14.00	92.00	97.33	97.33	96.66	45.33	60.00
DB_4_ -ExCuSe - set XII (524 images)	$\bar{d}$ **(px)**	32.72	5.35	4.46	6.80	31.16	32.82	23.81	8.69
	***σ_d_* (px)**	76.70	10.00	10.90	15.69	72.87	76.37	60.10	23.40
	***DR*_5_ (%)**	71.56	83.77	80.34	77.48	71.94	70.61	67.93	63.54

**Table 2:** Mean value and standard deviation of the Euclidean distance and *DR*_50_ for all tested PDAs in the scene image.

PDA (30 Records/PDA)	CENT	CHT	EHT	ExCuSe	LSFE	PROJ	RANSAC	Starburst
$\bar{d}$ **(px)**	29.90	25.87	27.67	33.72	30.34	30.13	33.31	44.76
***σ* (px)**	20.34	17.60	18.88	48.72	19.85	20.94	23.52	31.05
***DR*_50_ (%)**	90.28	91.39	88.86	85.32	87.10	86.95	84.46	66.86

**Table 3:** Mean value and standard deviation of the Euclidean distance for cluster evaluation and *CDIR*_50_ for all tested PDAs in the scene image.

PDA (30 Records/PDA)	CENT	CHT	EHT	ExCuSe	LSFE	PROJ	RANSAC	Starburst
$\bar{d_{c}}$ **(px)**	19.37	18.65	21.35	25.29	19.98	20.70	21.52	28.90
***σ_c_* (px)**	17.99	14.28	14.99	42.95	16.81	16.32	19.83	25.37
***CDIR*_50_ (%)**	4.64	4.07	5.35	8.02	5.20	5.95	6.97	13.03

**Table 4:** Running time of all tested algorithms.

PDA	CENT	CHT	EHT	ExCuSe	LSFE	PROJ	RANSAC	Starburst
**Running time (s)**	0.011	0.0541	0.0904	0.094	0.0121	0.0124	0.0193	0.0189

**Table 5:** Testing results obtained for the propose eye tracking interface used in eye typing application.

Statistical Indicators	Typing Speed (Characters/Min)	TER (%)	NCER (%)	CER (%)	MR
Overall mean value	20.74	3.55	0.60	2.95	11.13
Standard deviation	2.08	2.91	1.22	2.20	1.05
